# Improvement in Smoothness of Fermented Soymilk Yogurt-Mimic by Effective Use of Applicable Lactic Acid Bacteria Strains

**DOI:** 10.3390/foods14183235

**Published:** 2025-09-18

**Authors:** Wei Fu, Akio Kobayashi, Hiroyuki Yano

**Affiliations:** 1 Institute of Food Research, National Agriculture and Food Research Organization, Tsukuba 305-8642, Japan; yano.hiroyuki267@naro.go.jp; 2 Research Center for Agricultural Information Technology, National Agriculture and Food Research Organization, Tsukuba 305-8642, Japan; kobayashi.akio544@naro.go.jp

**Keywords:** soymilk yoghurt, lactic acid bacteria, smoothness

## Abstract

Fermented soymilk yogurt has been produced rapidly in recent years due to its health benefits and the growing demand for plant-based foods to address trends in sustainable development goals. This study investigated the smoothness and quality of soymilk yogurt-mimics fermented by four strains of lactic acid bacteria (LAB) through image processing, physicochemical properties, and taste analysis. By comparing the primary fermentation products of the four strains and the secondary fermentation products produced using the primary fermentation products with different refrigeration time as passage cultures, it was found that control strain could not produce fermented soymilk yogurt-mimic with good smoothness via technical improvement, while the AL3G1 strain, AL21D1 strain, and AL28A1 strain, which were used to produce the secondary fermentation products fermented using their primary fermentation products refrigerated for four days, exhibited relatively good smoothness, and superior rheological properties and flavor quality. This study aims to contribute an approach to improving the smoothness of fermented soymilk yogurt-mimic by effective use of applicable LAB strain and proper fermentation conditions. It will be beneficial to meet the strong demand for fermented soymilk product commercialization.

## 1. Introduction

Yogurt is a widely consumed fermented food product worldwide. Traditionally, yogurt is made by fermenting dairy milk with lactic acid bacteria (LAB). During the fermentation process, LAB breaks down lactose to form lactic acid, which gives yogurt its distinctive flavor, and lactic acid also causes a decrease in pH, which induces the aggregation of milk proteins to form a gel-like appearance [1]. Additionally, yogurt offers high-quality protein, calcium, and vitamins, and is suitable for people who are lactose intolerant [2]. However, a decline in intake of animal-based milk products has recently raised concerns because of the rising emergencies ranging from health aspects (such as milk allergy and cholesterol issues) to lifestyle choices, including vegetarians and vegans [3,4,5]. There is an urgent need to increase supply of plant-based milk alternatives that have the potential to replace animal-based milk in food making. The plant-based milk alternatives are facing the challenge of developing products that are as close as possible to dairy milk products in terms of taste, nutritional value, and appearance. It is worth mentioning that soymilk is a convenient and healthy food material that has been the most widely consumed plant-based milk alternative due to soybean’s remarkable nutrient profile, and it is the unique plant-based milk alternative that supplies the most value close to dairy milk. Soymilk is considered as a water extract of soybean, which is homogenized in water together with emulsifiers and stabilizers to result in colloidal suspensions of distribution in range of 5–20 µm, which is close to dairy milk in appearance and consistency [6]. Soybean not only contains high-quality protein comprising almost all the essential amino acids that correspond to those required for humans, with high digestibility of 92–100%, but is also rich in lipids and carbohydrates [7]. It also contains large amounts of polyunsaturated fatty acids, and is a rich source of vitamins, isoflavones, and minerals such as calcium, potassium, magnesium, iron, zinc, and copper, but no cholesterol. For these reasons, soymilk has also been utilized to produce many soy products like tofu, yuba, and even vegetarian chicken to enhance its use [8,9]. Additionally, commercial soymilk yogurt has grown rapidly in recent years due to its health benefits and the growing demand for plant-based foods to address trends in sustainable development goals [10]. Soymilk yogurt making follows a proper regimen due to characteristics of soy proteins. As with traditional dairy yogurt, fermentation is considered an effective and easy way to produce soymilk yogurt [11]. The fermentation process is essentially accomplished by microbial proliferation that involves the use of bacterial cultures. Generally, LAB is a common starter culture used in soymilk yogurt production. LAB can convert sugar of soymilk to lactic acid, which in turn lowers the pH of soymilk to form a yogurt-like solid with distinct and rich fermentation flavor. Therefore, soymilk yogurt making can be viewed as a sour curding process induced by LAB, and hence the texture of the sour gel varies depending on the type of LAB cultures used [12,13].

In general, the texture of soymilk yogurt is rough due to the high hardness and low water-holding capacity (WHC) that seriously hinder soymilk yogurt’s commercialization [14]. Recently, commercial soymilk yogurts are expected to have similar textural characteristics to traditional dairy milk yogurts to achieve popularity. Smoothness is a defining characteristic of high-quality yogurt that significantly impacts consumer acceptance, describing a uniform and creamy texture without graininess or excessive firmness. In the past literature, there have been reports of improved smoothness of soymilk yogurt with the addition of low-acyl gellan gum and cassava starch [15,16]. However, it is not easy to accept by consumers concerned about food additives, and there is a need to ascertain the digestibility of the mixed plant-based ingredients to ensure their contribution to protein intake. Furthermore, some researchers focused on the effect of extracellular polysaccharide (EPS) on the texture of soymilk yogurt. The use of LAB with high EPS production can provide better smoothness for soymilk yogurt via improving the rheology, water-holding capacity, and mouthfeel properties [17,18,19]. Although EPS is well known to enhance rheological and textural properties of yogurt and can be recognized as a safe ingredient in various food applications, the composition and quantity of EPS produced by LAB vary depending on factors such as LAB strains, pH, temperature, minerals, and carbon source. In addition, the cost of EPS is also taken into account for practical applications [11,20].

This study aims to provide a solution to improve the smoothness of fermented soymilk yogurt-mimic without the use of any additives or EPS, which is achieved by effectively utilizing applicable LAB strains and optimizing their fermentation conditions. Specifically, four LAB strains of *Lacticaseibacillus paracasei*, *Loigolactobacillus coryniformic*, *Weissella cibaria*, and *Latilactobacillus sakei* are selected for primary fermentation of soymilk. In addition, the primary fermented products are stored under refrigeration at different times. Moreover, the primary fermented products with different refrigeration storage times are used as passage cultures for secondary fermentation of soymilk. By analyzing the physicochemical properties, including pH and rheological properties and flavor quality of the primary and secondary fermentation products, the contributions of the four LAB strains to the fermented soymilk yogurt-mimic are compared and evaluated, leading to the identification of applicable strains and their fermentation conditions that contribute to improving the smoothness of fermented soymilk yogurt-mimic. It is hoped this study will be beneficial to meet the strong demand for the commercialization of fermented soymilk products.

## 2. Materials and Methods

### 2.1. Materials and Lactic Acid Bacteria Strains

Commercial plain soymilk (10% soy solids, 5.1 g protein, 3.1 g fat, 1.8 g carbohydrates, 0 mg cholesterol, 0 g salt equivalent, and 31 mg isoflavones per 100 mL) was used to ferment into a yogurt-mimic. The LAB strains of control (*Lacticaseibacillus paracasei*), AL3G1 (*Loigolactobacillus coryniformis*), AL21D1 (*Weissella cibaria*), and AL28A1 (*Latilactobacillus sakei*) were selected from the “NARO Lactic Acid Bacteria Collection”, which is independently integrated and centrally managed by the National Agriculture and Food Research Organization (NARO). Approximately 3000 strains in the “NARO *Lactobacillus* Collection” are derived from food sources such as crops and fermented foods.

### 2.2. Preparation of LAB Cultures and Fermented Soymilk Yogurt-Mimic

The LAB strains were stored at −80 °C. Each strain was inoculated aseptically into 5 mL of MRS broth medium under aseptic conditions and incubated at 30 °C for 24 h under static conditions for both pre-cultivation and main cultivation. The cultivated MRS medium (10^8^~10^9^ cfu/mL) was centrifuged at 25 °C, 5000× *g* to collect the bacteria for 10 min, and then the bacteria were washed once with 0.85% saline solution and resuspended in an equal volume of saline solution to prepare the lactobacilli culture. The LAB culture of each strain was added into commercial plain soymilk at a concentration of 0.1% *v*/*v*, fermenting at 30 °C for 24 h to produce the original yogurt (Y0-1), which was considered as primary fermentation. Additionally, Y0-1 was refrigerated at 4 °C for 1 day (Y1-1), 4 days (Y4-1), and 7 days (Y7-1), which were analyzed to investigate the effect of refrigeration on the primary fermentation products. Furthermore, the primary fermentation products were, respectively, added at a concentration of 10% *w*/*w* into commercial plain soymilk as passage cultures, fermenting at 30 °C for 24 h, to produce the secondary fermentation products, which were then compared with the primary fermentation products for investigating the effect of the primary fermentation product as a passage culture on the yogurt-mimic. As the samples, the primary fermentation products of Y0-1, Y1-1, Y4-1, and Y7-1 and the secondary fermentation products of Y0-2, Y1-2, Y4-2, and Y7-2, which were fermented by Y0-1, Y1-1, Y4-1, and Y7-1, respectively, were analyzed.

### 2.3. Smoothness Observation

Each sample was stirred by a spoon in a Petri dish (90 mm in diameter, 15 mm in height), and the smoothness was evaluated in terms of the unevenness of the surface. A digital camera (EX-100, CASIO Computer Co., Ltd., Tokyo, Japan) was used above the Petri dish to take photographs, and ImageJ (Version 1.54g) was used to detect the photographs. The percentage ratio of uneven and irregular granular area relative to the total area was calculated. The image was processed at a type of 8-bit and radius of 2.0 pixels. The bar was 10 mm.

### 2.4. Physicochemical Analysis

The physicochemical properties of each sample were analyzed based on pH, titratable acidity, and rheological properties. The pH of each sample was monitored by a pH meter (LAQUA F-72, HORIBA Ltd., Kyoto, Japan). The titratable acidity was determined by titrating with 0.1 mol/L NaOH solution, and the value was calculated using the following formula as lactic acid (%). A dynamic viscoelasticity test was performed by a rheometer (HAAKE MARS iQ Air Rotational Rheometer, Thermo Fisher Scientific K.K., Tokyo, Japan) for the rheological properties. The well-stirred samples were put on the Peltier plate, and a parallel plate was positioned to the joint surface of the samples, forming a space of diameter 35 mm and a gap of 1 mm. The Job Manager Software (HAAKE RheoWin 4.87.0001 Job Manager) was used to confirm the changes in viscoelasticity characteristics by a program of Osc stress sweep. The conditions were set as follows: the shear stress (τ) was set from 0.01 Pa to 10 Pa, the frequency (f) was 1 Hz, and the temperature was 30 °C. Each sample was measured at least 3 times for technical replicates. The Data Manager Software (HAAKE RheoWin 4.87.0001 Data Manager) was used for analyzing storage modulus (G′), loss storage (G″), and loss factor of tan (δ).

Titratable acidity (%) = 9 × (V/W) × factor × (1/10)

V is the volume (mL) of the titration value, and W is the weight (g) of the sample. Factor (20 °C) is 1.000 according to the indicator method.

### 2.5. Taste Sensor Analysis

Taste sensor analysis was carried out in the Japan Food Research Laboratory (JFRL, Tokyo, Japan), which is one of the world’s largest testing service providers, according to its standard methods. TS-5000Z taste recognition device (Intelligent Sensor Technology, Inc., Kanagawa, Japan) and five types of sensors, including CA0 (sourness), AAE (umami), C00 (bitterness), AE1 (astringency), and CT0 (saltiness) for food evaluation, were used to analyze taste. The test solutions were prepared by filtering the well-stirred and uniform samples after dilution with an equal amount of distilled water. The measurement potential in the reference solution of each sensor was set to zero, and the difference in potential between the test solution and the reference solution was considered as the initial taste, and when the reference solution was used to wash the sensor and its potential was measured again, the difference in potential was considered as the aftertaste. The aftertaste of umami, bitterness, and astringency was measured by the taste substances remaining in the AAE, C00, and AE1 sensors, respectively. The potential difference was corrected and numerically converted using software (TS-5000Z Analysis Application Ver3.0.0), and the evaluation of each taste was displayed as a radar chart.

### 2.6. Statistical Analysis

According to IBM SPSS Statistics Ver. 28.0.1.0 (142) software, one-way analyses of variance were used to test the differences in physicochemical properties and rheological properties. The significance was defined as *p* < 0.05.

## 3. Results

### 3.1. Smoothness of Fermented Soymilk Yogurt-Mimic

**Figure 1:**
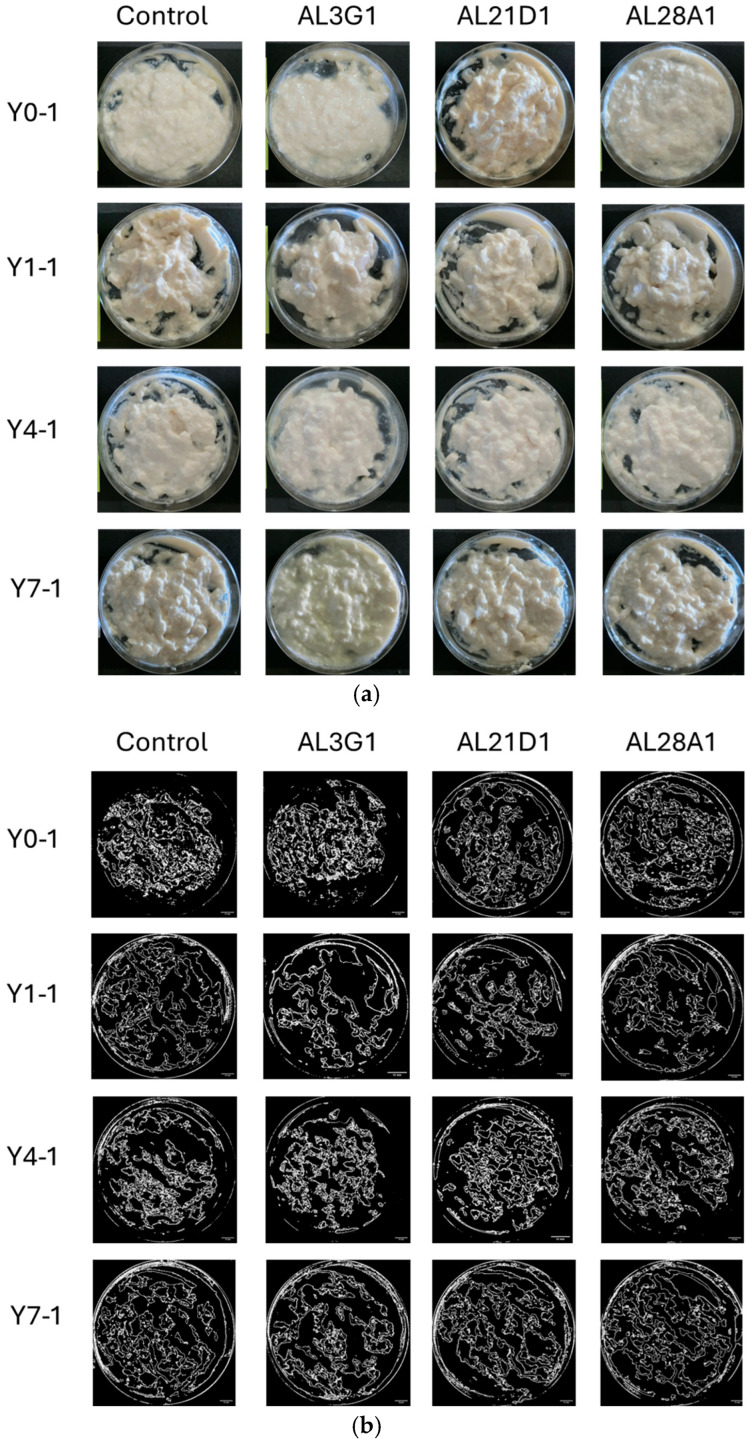
(**a**) Photos of the primary fermentation products after stirring. The horizontal direction represents differences in bacterial strains, and the vertical direction represents differences in refrigeration time. Y0-1 is non-refrigerated, Y1-1 is refrigerated for 1 day, Y4-1 is refrigerated for 4 days, and Y7-1 is refrigerated for 7 days. (**b**) ImageJ processing photos of the primary fermentation products after stirring. The horizontal direction represents differences in bacterial strains, and the vertical direction represents differences in refrigeration time. Y0-1 is non-refrigerated, Y1-1 is refrigerated for 1 day, Y4-1 is refrigerated for 4 days, and Y7-1 is refrigerated for 7 days. The bar is 10 mm.

**Figure 2:**
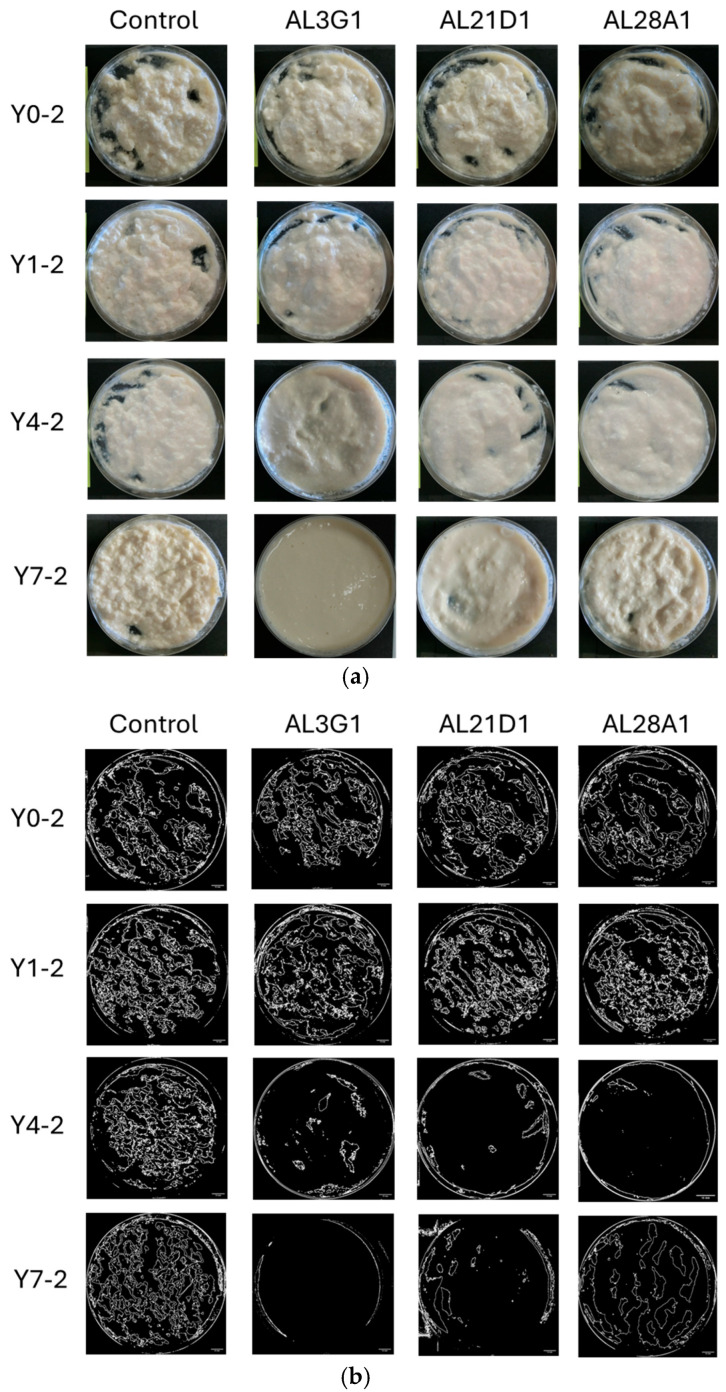
(**a**) Photos of the secondary fermentation products after stirring. The horizontal direction represents differences in bacterial strains, and the vertical direction represents differences when added with various primary fermentation products for passage cultures. Y0-2 is fermented using the primary fermentation product of non-refrigeration, Y1-2 is fermented using the primary fermentation product refrigerated for 1 day, Y4-2 is fermented using the primary fermentation product refrigerated for 4 days, and Y7-2 is fermented using the primary fermentation product refrigerated for 7 days. (**b**) ImageJ processing photos of the secondary fermentation products after stirring. The horizontal direction represents differences in bacterial strains, and the vertical direction represents differences when added with various primary fermentation products for passage cultures. Y0-2 is fermented using the primary fermentation product of non-refrigeration, Y1-2 is fermented using the primary fermentation product refrigerated for 1 day, Y4-2 is fermented using the primary fermentation product refrigerated for 4 days, and Y7-2 is fermented using the primary fermentation product refrigerated for 7 days. The bar is 10 mm.

Figure 1a shows the status of smoothness in the primary fermentation products. The smoothness of yogurt-mimics was assessed based on the status of unevenness and granularity of the surface after stirring. To objectively reflect the differences, ImageJ was used for image processing and numerical calculation of the roughness area. As a result, it was shown that all primary fermentation products following different refrigeration times, which were fermented by the four LAB strains, exhibited uneven and irregular granular appearance in Figure 1b. Additionally, the ratio of roughness (uneven and irregular granular) area was shown in Table 1. In the primary fermentation products, including Y0-1, Y1-1, Y4-1, and Y7-1, the roughness area accounted for approximately 24–46% of the total surface, indicating a certain degree of roughness. From the above, it was revealed that the primary fermentation products did not exhibit smoothness regardless of variations in strains (control, AL3G1, AL21D1, and AL28A1) or refrigeration time. On the other hand, when the primary fermentation products (Y0-1, Y1-1, Y4-1, Y7-1) were used as passage cultures for secondary fermentation, as shown in Figure 2a,b, although the smooth appearance of all strains’ secondary fermentation products in Y0-2 and Y1-2 was not exhibited, there were obvious improvement on smoothness of Y4-2 and Y7-2 in AL3G1, AL21D1 and AL28A1. It was shown that the secondary fermentation products of control strains Y4-2 and Y7-2 still exhibited unevenness and granularity, while those of the other three strains in Y4-2 were obviously improved. Further, the ratio of roughness area of AL3G1, AL21D1, and AL28A1 in Y4-2 accounted for lower proportions of 7.6%, 5.2%, and 2.3%, respectively (Table 1). From the above, it was revealed that the primary fermentation products of AL3G1, AL21D1, and AL28A1, which were refrigerated for 4 days, had the capacity to improve the smoothness of the soymilk yogurt as a passage culture, except for the control strain. In other words, it is possible to produce high-quality soymilk yogurt with a smooth texture by appropriately and effectively utilizing AL3G1, AL21D1, and AL28A1. In the past literature, technical methods for improving the smoothness of soymilk yogurt mainly focus on using polysaccharides to improve yogurt quality and combining some special raw materials. Grasso et al. investigated sensory properties of six commercial plant-based yogurts that had good sensory characteristics, attributing this to the use of hydrocolloids in the plant-based yogurt [21]. Furthermore, ingredients like low acyl gellan gum, starch, and banana powder were used to make an improvement in order to give soymilk yogurts a smooth texture [15,16,22]. Additionally, the mouthfeel properties of soy yogurt products could be optimized by regulating fermentation using EPS-producing probiotic strains. However, the composition and quantity of EPS produced by LAB strains may vary depending on factors such as LAB strains, pH, temperature, minerals, vitamin availability, and carbon source [11,17,18]. In this study, the smoothness of soymilk yogurt was improved by passage cultures of strains rather than additives or EPS, which requires more stringent fermentation conditions. This could reduce time and costs in future production applications of soymilk yogurt. In addition, improving the smoothness of soymilk yogurt-mimic was assessed solely through image processing to distinguish the effectiveness of selected strains and fermentation methods in this study. For further research, more quantitative methods such as texture profile analysis (TPA) or back extrusion will be essential when conducting more in-depth analyses of smoothness improvement.

### 3.2. Physicochemical Properties of Fermented Soymilk Yogurt-Mimic

#### 3.2.1. pH Values of Fermented Soymilk Yogurt-Mimic

**Figure 3:**
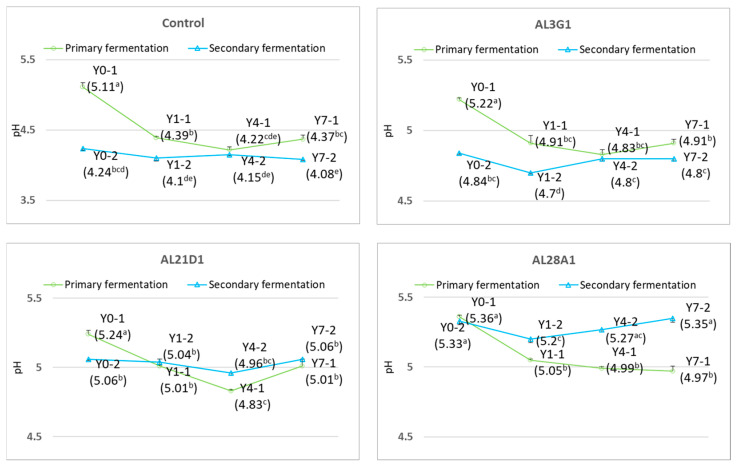
pH changes in fermented soymilk yogurt-mimics in primary fermentation and secondary fermentation. pH values with different superscripted letters are significantly different (*p* < 0.05). Y0-1 is non-refrigerated, Y1-1 is refrigerated for 1 day, Y4-1 is refrigerated for 4 days, and Y7-1 is refrigerated for 7 days. Y0-2 is fermented using Y0-1, Y1-2 is fermented using Y1-1, Y4-2 is fermented using Y4-1, and Y7-2 is fermented using Y7-1.

Figure 3 shows pH changes in the primary fermentation products fermented by four LAB strains when refrigerated for different times and the secondary fermentation products fermented using the primary fermentation products for different refrigerated times. In the primary fermentation products, the pH values of primary fermentation products of all types of strains decreased significantly after refrigeration compared to the primary fermentation products that were not refrigerated. This result was consistent with findings from the other researchers. Hati et al. also reported that the pH of fermented sogurt decreased significantly with an increase in storage time [23]. Compared to the control strain, whose pH decreased from 5.11 to 4.22, the pH decreases in the other three strains were relatively smaller, with the pH values being 5.22 to 4.83, 5.24 to 4.83, and 5.36 to 4.97, respectively. Further, the pH of the primary fermentation products of the control strain and AL21D1 strain decreased significantly after refrigeration for 1 day and 4 days, but the pH of the primary fermentation product of the AL21D1 strain slightly increased after refrigeration for 7 days. In contrast, the pH of the primary fermentation products of the AL3G1 strain and AL28A1 strain did not change significantly after refrigeration for 1 day, 4 days, or 7 days. In the past literature, the pH value of the fermented soy yogurt remained mostly unchanged during the storage at 4 °C, which was explained as the stability in pH value was presumably due to lower activity of the starter culture during refrigerated storage [24]. Therefore, no obvious fluctuation in pH could be considered as refrigeration restricted the activity of AL3G1 and AL28A1 in this study. In addition, all four strains showed a tendency to reach their lowest pH values at 4 days of refrigeration. On the other hand, when using the primary fermentation products as passage cultures for secondary fermentation, the secondary fermentation products of the control and AL3G1 showed a tendency toward lower pH compared to the primary fermentation products, especially the secondary fermentation products of Y0-2 and Y1-2, which were fermented using Y0-1 and Y1-1, respectively, and had a significant decrease in pH value. The pH of Y0-2 of AL21D1 showed a significant decrease, while the refrigerated primary fermentation products (Y1-1, Y4-1, and Y7-1) had no significant effect on the pH of the secondary fermentation products of AL21D1. The refrigerated primary fermentation products of AL28A1 caused a significant increase in the pH of the secondary fermentation products. Considering the pH of the secondary fermentation products only, regardless of the refrigeration time of the primary fermentation products, the pH values of the secondary fermentation products in each strain remained stable with minimal variation or no variation at all. The primary fermentation products of the control and AL21D1 did not cause significant pH changes in the secondary fermentation products. The primary fermentation products of AL3G1 and AL28A1 after 1 day of refrigeration caused a slightly significant decrease in the pH of secondary fermentation products, but the primary products after 4 days and 7 days of refrigeration brought the pH of secondary fermentation products back to a level close to that of those fermented using the primary fermentation products without refrigeration. From the above, although the pH of the primary fermentation products of each strain showed significant changes after refrigeration, when using the primary fermentation products with different refrigeration times as passage cultures for secondary fermentation, the pH did not undergo significant changes in the secondary fermentation products, which meant that the improvement in smoothness of AL3G1, AL21D1, and AL28A1 in Y4-2 comparing to their Y0-2 and Y1-2 was not caused by pH changes (mentioned earlier in Figure 2). In addition, there was no significant improvement in the smoothness of the secondary fermentation products of the control, because the pH of the secondary fermentation products of the control was between 4.24 and 4.08, while the pH of the secondary fermentation products of other strains remained between 4.7 and 5.3. This may be because an excessively low pH is not conducive to improving the smoothness of yogurt. It was demonstrated that pH is an important quality parameter of yogurt formulations that affects its coagulation. The sharply decreased pH values were achieved with the increasing fermentation time [25]. LAB produces acid in the fermentation process of soy yogurt, which leads to the aggregation of proteins in the soymilk to form the network structure, and the faster the pH decreases, the stronger the aggregation [26]. In this study, the control strain showed the best performance in acid production. Although the refrigeration limited its activity as a starter culture, the lower pH formed during the fermentation process promoted stronger protein aggregation and consistency, leading to a firmer, less smooth texture.

#### 3.2.2. Rheological Properties of Fermented Soymilk Yogurt-Mimic

**Figure 4:**
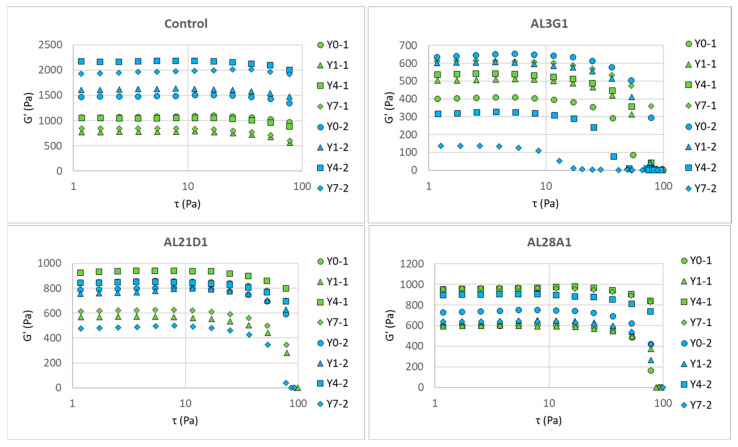
G′ (storage modulus) change in fermented soymilk yogurt-mimics of different LAB strains between primary fermentation and secondary fermentation in a shear stress sweep. Green color represents the primary fermentation, and blue color represents the secondary fermentation. Y0-1 is non-refrigerated, Y1-1 is refrigerated for 1 day, Y4-1 is refrigerated for 4 days, and Y7-1 is refrigerated for 7 days. Y0-2 is fermented using Y0-1, Y1-2 is fermented using Y1-1, Y4-2 is fermented using Y4-1, and Y7-2 is fermented using Y7-1.

**Figure 5:**
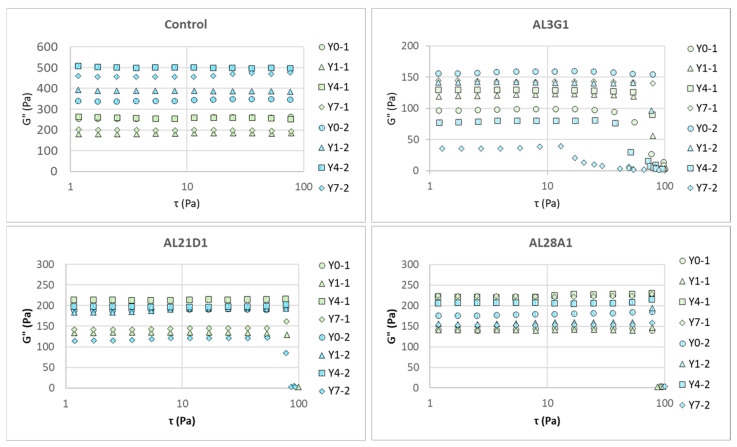
G″ (loss modulus) change in fermented soymilk yogurt-mimics of different LAB strains between primary fermentation and secondary fermentation in a shear stress sweep. Green color represents the primary fermentation, and blue color represents the secondary fermentation. Y0-1 is non-refrigerated, Y1-1 is refrigerated for 1 day, Y4-1 is refrigerated for 4 days, and Y7-1 is refrigerated for 7 days. Y0-2 is fermented using Y0-1, Y1-2 is fermented using Y1-1, Y4-2 is fermented using Y4-1, and Y7-2 is fermented using Y7-1.

Figure 4 shows G′ (storage modulus) changes in the fermented soymilk yogurt-mimics of different LAB strains between primary fermentation and secondary fermentation in the dynamic viscoelasticity test following a shear stress sweep, and Figure 5 shows G″ (loss modulus) changes. G′ is an indicator of material’s elastic properties and is used to evaluate its hardness. G″ is an indicator of material’s viscous properties and can be used to evaluate its flow characteristics to some extent. Table 2 shows the initial G′ and G″ measured at 1 τ in Pa, which could reveal the hardness and flow characteristics of the samples. In the primary fermentation products, the G′ of the fermentation products from the control and AL21D1 did not exhibit any regular changes as the refrigeration time increased, while the G′ in AL3G1 and AL28A1 exhibited an increasing trend with prolonged refrigeration time. In the secondary fermentation products, it was shown that the longer the refrigeration time of the first fermentation product, the lower the G′ of the second fermentation product in AL3G1, while the G′ of the second fermentation products in the other three strains showed no regular changes (Figure 4, Table 2). Additionally, compared to the G′ values of less than 1000 Pa in AL3G1, AL21D1, and AL28A1, the control strain had a significantly higher value around 2000 Pa, which may be related to the lower pH of the secondary fermentation product in the control strain. A lower pH indicates higher acidity, which is more likely to cause protein aggregation, as mentioned in the other studies [14,26]. Also, a higher G′ value indicates a greater storage elasticity, which could be considered as a reason for the unevenness and granularity observed in the fermentation product from the control.

**Figure 6:**
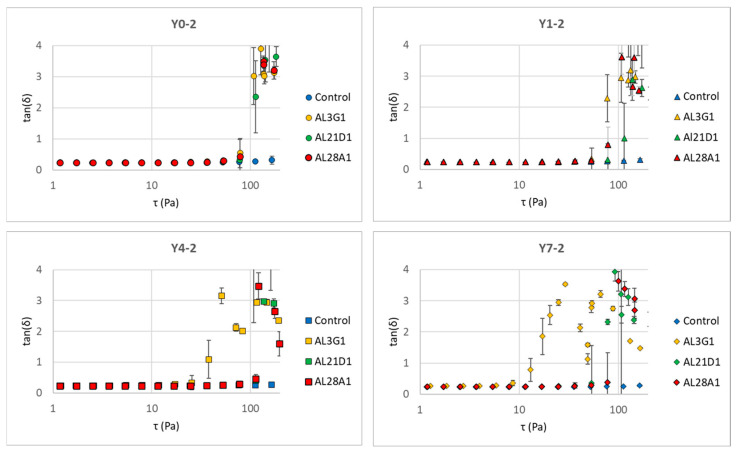
tan (δ) change in secondary fermentation products fermented using primary fermentation products with different refrigeration times for different LAB strains in a shear stress sweep. Y0-2 is fermented using Y0-1, Y1-2 is fermented using Y1-1, Y4-2 is fermented using Y4-1, and Y7-2 is fermented using Y7-1.

The same trend could also be found in G″, but it is worth noting that the changes in G″ tend toward stability in all strains, while the G′s of AL3G1, AL21D1, Al28A1 exhibited an acute decline in storage elasticity as the shear stress increased, indicating that the fermentation products of these three strains tend toward a more fluid status under shear stress. This could also be verified through the tan value. Whether the material tends toward fluidity depends on the tan value. The tan value is calculated as G″/G′. The tan value is another important indicator of a material’s rheological properties. When the tan value approaches 0, it indicates a tendency toward elasticity, while a higher tan value indicates a more viscous and less elastic material, which corresponds to a smoother and softer texture with increased fluidity. Yang et al. also reported that the storage modulus (G′) of the plant-based yogurt mimic samples increased faster than the loss modulus (G″) during the acidification process that forms the gel, indicating that yogurt gel had high elastic properties [27]. In this study, when G″ kept stable, the acute decrease in G′ caused the increased tan value, indicating a tendency toward viscosity and fluidity. Figure 6 shows the changes in tan values of the secondary fermentation products fermented using the primary fermentation products with different refrigeration times. Regardless of the primary fermentation products used for secondary fermentation, the tan values of AL3G1, AL21D1, and AL28A1 were higher than those of the control, which was considered to be associated with the improved smoothness of the secondary fermentation products in these three strains.

### 3.3. Taste Sensor Analysis of Fermented Soymilk Yogurt

**Figure 7:**
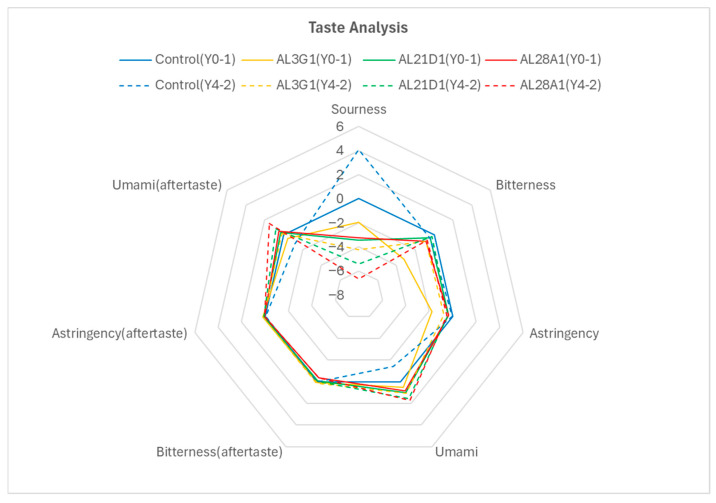
Taste sensor analysis of the non-refrigerated primary fermentation products fermented by the LAB strain and the secondary fermentation products fermented using the primary fermentation product refrigerated for 4 days. The solid line is Y0-1, and the dotted line is Y4-2.

As a yogurt, taste and flavor are also important factors in assessing its quality. To demonstrate that the fermented soymilk yogurts with improved smoothness in this study also possess good taste and flavor quality, a taste analysis was conducted on the primary fermentation products of Y0-1 and the secondary fermentation products of Y4-2 with good smoothness (Figure 7). In Y0-1, compared to the product fermented by the control, the sourness of AL3G1, AL21D1, and AL28A1 showed reduced values, which were consistent with the previously mentioned lower pH in the control’s primary fermentation product (Figure 3). Further, the bitterness and astringency of AL3G1, AL21D1, and AL28A1 were improved. Surprisingly, the umami flavor of these three strains was enhanced, both in terms of their inherent taste and aftertaste. Mu et al. reported that the umami of the walnut and purple rice fermented yogurts was superior to dairy-based yogurt because the output of umami is inhibited by calcium, especially in calcium-rich samples such as dairy-based yogurt and cheese [28]. On the other hand, compared to Y0-1, the control in Y4-2 showed a trend toward increased sourness, which also aligned with the acidity results (Table 3). However, the sourness of AL3G1, AL21D1, and AL28A1 in Y4-2 was reduced compared to Y0-1. Although this did not objectively align with the acidity results, it could be considered within the margin of error. This was because the acidity changes between the primary fermentation products and the secondary fermentation products of AL3G1, AL21D1, and AL28A1 were limited (0.2% in acidity, Table 3). Further, the taste analysis in this study was based on a pH of 4.5 as the benchmark for sourness perception, and all the samples (control, AL3G1, AL21D1, and AL28A1) exhibited negative potential difference values (Table 4), which were significantly weaker than the sourness expression of the taste threshold, and hence more difficult to discern at the human perception level. Regarding the other taste aspects, compared to Y0-1, there were no significant changes in bitterness and astringency in all the strains of Y4-2, but there was a trend toward enhanced umami flavor in AL3G1, AL21D1, and AL28A1. Taste is a complex sensation perceived by taste buds, and the strengths and interactions of various tastes create a complex perceptual system. Therefore, it is inaccurate to assess a specific taste in isolation. Additionally, even the absence of a prominent taste sensation in taste analysis does not necessarily mean that the taste is absent. It is crucial to account for the sensory situation of the raw material itself. In this study, inconspicuous bitterness and astringency, and prominent umami may reflect the richness of soy flavor and intense fragrance in the products.

## 4. Conclusions

This study evaluated the smoothness of the primary and secondary fermentation products fermented by four LAB strains and analyzed the physicochemical properties and flavor quality in conjunction with smoothness. It was found that AL3G1, AL21D1, and AL28A1 can improve the smoothness of fermented soymilk yogurt-mimic without the use of any additives through reasonable refrigeration methods, enhancing umami quality and reducing sourness of yogurt-mimic. Compared to improving the smoothness of yogurt by use of additives or high-cost exopolysaccharides, the approach of improving the smoothness of LAB fermented yogurt-mimic through processing techniques in this study is expected to be applied and popularized in future actual production.

## 5. Patents

This application was filed with the Japan Patent Office on 25 February 2025 for technical details regarding the production of soymilk yogurt with good smoothness by fermentation using new lactic acid bacteria. Application No.: Patent Application 2025-028426.

## Figures and Tables

**Table 1:** Ratio of roughness area of fermented soymilk yogurt-mimics.

Samples	Roughness Area (%)			
	Control	AL3G1	AL21D1	AL28A1
Y0-1	43.3%	36.0%	40.5%	30.1%
Y1-1	43.4%	24.8%	41.4%	38.8%
Y4-1	41.2%	24.7%	40.0%	38.2%
Y7-1	41.4%	42.6%	46.2%	44.1%
Y0-2	35.8%	39.3%	35.7%	41.5%
Y1-2	39.2%	30.1%	39.8%	33.0%
Y4-2	29.9%	7.6%	5.2%	2.3%
Y7-2	51.2%	0.0%	8.4%	17.4%

Note: The ratio values are calculated from ImageJ processing data.

**Table 2:** G′ and G″ (1 τ in Pa) of fermented soymilk yogurt-mimics.

Samples	G′ (Pa)			
	Control	AL3G1	AL21D1	AL28A1
Y0-1	1047 ^a^	402 ^f^	804 ^b^	585 ^g^
Y1-1	734 ^b^	492 ^g^	585 ^g^	545 ^g^
Y4-1	1052 ^a^	589 ^g^	938 ^j^	962 ^j^
Y7-1	806 ^b^	616 ^g^	656 ^g^	937 ^j^
Y0-2	1394 ^c^	714 ^b^	875 ^j^	680 ^g^
Y1-2	1724 ^d^	656 ^g^	801 ^b^	629 ^g^
Y4-2	2193 ^e^	308 ^h^	872 ^j^	918 ^j^
Y7-2	1782 ^d^	141 ^i^	445 ^f^	606 ^g^
	G″ (Pa)			
	Control	AL3G1	AL21D1	AL28A1
Y0-1	250 ^a^	97 ^g^	185 ^i^	138 ^i^
Y1-1	175 ^b^	119 ^h^	139 ^i^	129 ^i^
Y4-1	256 ^c^	141 ^i^	216 ^l^	224 ^l^
Y7-1	189 ^d^	142 ^i^	150 ^i^	218 ^l^
Y0-2	326 ^e^	171 ^i^	208 ^l^	163 ^i^
Y1-2	416 ^f^	157 ^i^	192 ^i^	155 ^i^
Y4-2	515 ^f^	75 ^j^	206 ^l^	215 ^l^
Y7-2	425 ^f^	36 ^k^	106 ^h^	145 ^i^

Note: The value represents G′ or G″ at shear stress of 1. Values with different superscripted letters in the G′ group or G″ group are significantly different (*p* < 0.05).

**Table 3:** Titratable acidity of fermented soymilk yogurt-mimics.

Samples	Titratable Acidity (%)			
	Control	AL3G1	AL21D1	AL28A1
Y0-1	0.5	0.42	0.43	0.4
Y1-1	0.66	0.55	0.49	0.46
Y4-1	1	0.58	0.62	0.5
Y7-1	0.92	0.56	0.54	0.52
Y0-2	1.02	0.59	0.53	0.41
Y1-2	1.08	0.62	0.57	0.45
Y4-2	1.09	0.61	0.58	0.41
Y7-2	1.12	0.6	0.55	0.4

Note: Acidity is determined by titrating with 0.1 mol/L NaOH solution. The value of titratable acidity is represented as lactic acid (%).

**Table 4:** Potential differences for each taste in fermented soymilk yogurt-mimics of Y0-1 and Y4-2.

	Y0-1						
	Sourness	Bitterness	Astringency	Umami	Bitterness (aftertaste)	Astringency (aftertaste)	Umai (aftertaste)
Control	−19.52 ^a^	5.58 ^a^	2.80 ^a^	6.35 ^a^	0.32 ^a^	0.18 ^a^	4.14 ^ab^
AL3G1	−21.47 ^b^	2.35 ^b^	1.03 ^b^	6.86 ^ab^	0.37 ^b^	0.36 ^b^	3.72 ^a^
AL21D1	−22.97 ^c^	5.18 ^c^	2.43 ^ac^	7.35 ^b^	0.25 ^c^	0.27 ^cd^	4.44 ^bc^
AL28A1	−22.75 ^c^	4.69 ^d^	2.44 ^ac^	7.20 ^b^	0.02 ^d^	0.24 ^c^	4.63 ^bc^
	Y4-2						
	Sourness	Bitterness	Astringency	Umami	Bitterness (aftertaste)	Astringency (aftertaste)	Umai (aftertaste)
Control	−15.83 ^d^	5.03 ^c^	2.41 ^ac^	5.78 ^c^	0.28 ^c^	0.18 ^a^	2.85 ^d^
AL3G1	−24.19 ^e^	4.58 ^d^	1.72 ^d^	8.24 ^d^	0.38 ^b^	0.36 ^b^	4.23 ^ab^
AL21D1	−25.37 ^f^	5.25 ^c^	2.09 ^cd^	8.75 ^de^	0.21 ^e^	0.30 ^d^	4.86 ^c^
AL28A1	−26.60 ^g^	4.75 ^d^	1.95 ^cd^	8.94 ^e^	0.08 ^f^	0.27 ^cd^	5.58 ^e^

Note: Values with different superscripted letters in the same column are significantly different (*p* < 0.05).

## Data Availability

The original contributions presented in the study are included in the article, further inquiries can be directed to the corresponding author.

## Abbreviations

The following abbreviations are used in this manuscript:

LAB	Lactic acid bacteria
WHC	Water-holding capacity
EPS	Extracellular polysaccharide
